# Movement related slow cortical potentials in severely paralyzed chronic stroke patients

**DOI:** 10.3389/fnhum.2014.01033

**Published:** 2015-01-15

**Authors:** Ozge Yilmaz, Niels Birbaumer, Ander Ramos-Murguialday

**Affiliations:** ^1^Institute of Medical Psychology and Behavioral Neurobiology, University of TuebingenTuebingen, Germany; ^2^Brain and Mind Studies Lab, Department of Psychology, Bahcesehir UniversityIstanbul, Turkey; ^3^Ospedale San Camillo, Istituto di Ricovero e Cura a Carattere ScientificoLido di Venezia, Italy; ^4^Health Technologies Department, TecnaliaSan Sebastian, Spain

**Keywords:** stroke, movement related slow cortical potentials, EEG, movement preparation, intention to move

## Abstract

Movement-related slow cortical potentials (SCPs) are proposed as reliable and immediate indicators of cortical reorganization in motor learning. SCP amplitude and latency have been reported as markers for the brain's computational effort, attention and movement planning. SCPs have been used as an EEG signature of motor control and as a main feature in Brain-Machine-Interfaces (BMIs). Some reports suggest SCPs are modified following stroke. In this study, we investigated movement-related SCPs in severe chronic stroke patients with no residual paretic hand movements preceding and during paretic (when they try to move) and healthy hand movements. The aim was to identify SCP signatures related to cortex integrity and complete paralysis due to stroke in the chronic stage. Twenty severely impaired (no residual finger extension) chronic stoke patients, of whom ten presented subcortical and ten cortical and subcortical lesions, underwent EEG and EMG recordings during a cue triggered hand movement (open/close) paradigm. SCP onset appeared and peaked significantly earlier during paretic hand movements than during healthy hand movements. Amplitudes were significantly larger over the midline (Cz, Fz) for paretic hand movements while contralateral (C4, F4) and midline (Cz, Fz) amplitudes were significantly larger than ipsilateral activity for healthy hand movements. Dividing the participants into subcortical only and mixed lesioned patient groups, no significant differences observed in SCP amplitude and latency between groups. This suggests lesions in the thalamocortical loop as the main factor in SCP changes after stroke. Furthermore, we demonstrated how, after long-term complete paralysis, post-stroke intention to move a paralyzed hand resulted in longer and larger SCPs originating in the frontal areas. These results suggest SCP are a valuable feature that should be incorporated in the design of new neurofeedback strategies for motor neurorehabilitation.

## Introduction

Stroke occurs as a consequence of cardiovascular flow disturbances damaging neural networks in the brain. Following stroke, reorganization of cortical networks occurs (Grefkes et al., 2008; Ward, 2011). Reorganization consists among other consequences of enhanced neural activity of the healthy hemisphere (Chollet et al., 1991; Murase et al., 2004; Bashir et al., 2010).

Movement-related slow cortical potentials (SCPs) recorded with EEG can be divided into two main components: (a) potentials occurring during intention or anticipation of an upcoming movement which is also called the Bereitschaftspotential (BP) for self-paced movements (Barrett et al., 1986), (b) the motor potential (MP) occurring at the time of the execution (Deecke et al., 1969). A BP is a bilateral low frequency (0–5 Hz) negative shift (NS) occurring seconds before the movement onset. The MP peak rises primarily contralateral to the movement side around the onset of a voluntary movement (Birbaumer et al., 1991), which is also referred to as peak NS (Barrett et al., 1986; Shibasaki and Hallett, 2006). According to the excitation threshold theory, during the preparation of a movement SCPs serve as regulatory mechanisms that facilitate neuronal firing of the involved networks (Elbert and Rockstroh, 1987; Birbaumer et al., 1991).

Cortical activity preceding voluntary movements is well-documented (Kornhuber and Deecke, 1965). The main cortical generators of SCPs are the premotor cortices (PMC), supplementary motor areas (SMAs) and cingulate cortices (Deecke, 1987; Cui et al., 1999). There is emerging evidence that subcortical structures, particularly the basal ganglia, also contribute to movement preparation, execution and control. The thalamus is connected to cortex and both the basal ganglia and the cerebellar pathways and the role of these connections in movement preparation has recently been studied extensively in humans (Rektor, 2002; Paradiso et al., 2004). A lesion in any of these structures or connections (e.g., corticothalamic loop) could affect movement preparation and planning and should be reflected in SCPs, especially if motor recovery does not take place and chronic stage is reached.

It has been shown that the latency of SCP [i.e., the time between the onset of SCP and the movement onset recorded with electromyography (EMG)] indicates the preparation time of the required action, with longer the latencies indicating more complex is the motor tasks (Tarkka and Hallett, 1990; Lang et al., 1992). Repetitive simple movements, which do not require higher order cognitive pre-planning and preparation, are related to shorter SCP latency and smaller SCP amplitudes (Libet et al., 1982). It has been suggested that the amplitude of the negative peak may indicate the brain's computational “demand” to perform the movement (Libet et al., 1982; Lang et al., 1990; Simonetta et al., 1991; Libet, 1992).

Several groups have demonstrated altered SCP features after a brain disease or injury (Sasaki and Gemba, 1984; Cunnington et al., 1999). Kitamura et al. (1996), in a study involving two subcortical stroke patients performing synergistic movements of the paretic arm, report that the early SCP component remained bilateral as in healthy participants. However, MP was also distributed bilaterally as opposed to its dominant contralateral distribution in healthy individuals, indicating a stronger involvement of the healthy hemisphere during the movements of paretic side. Additionally, stroke patients' recovering motor function spontaneously shows an increase in fMRI BOLD activity toward the non-lesioned hemisphere when executing paretic hand movements. These are shifted back to ipsilesional areas once recovery takes place, reaching a “normal” bilateral activation with a peak at hand movement's contralateral hemisphere (Rossini et al., 2003; Murphy and Corbett, 2009). Green et al. (1999) offer support for these findings using multimodal neuroimaging methods with EEG and fMRI demonstrating that the intact hemisphere becomes more active after stroke in participants with varying degrees of recovery. Similar findings regarding contralesional activation were observed by other groups (Lang et al., 1988; Verleger et al., 2003; Jankelowitz and Colebatch, 2005). It has also recently been shown sensorimotor-rhythm (SMR) based EEG Brain-Machine-Interfaces (BMIs) can be used to recover motor function in chronic severely paretic stroke patients (Ramos-Murguialday et al., 2013). Furthermore, SCPs have been extensively used as features for neurofeedback and BMI control (Birbaumer et al., 1999).

In this study, we investigated the effects of cortex integrity and stroke severity on SCPs (i.e., neural reorganization in participants with severe hand weakness in the chronic stage). The final goal is to identify relevant features that can be used and optimized (e.g., toward normal potentials' characteristics) in SCP based BMI neurofeedback therapy for motor rehabilitation in paralyzed chronic stroke patients. We studied SCPs of 20 severely impaired chronic stroke patients, who suffered from subcortical and mixed (cortical and subcortical) lesions, during paretic and healthy hand movements. The aim was to investigate changes in SCPs in severe chronic stroke comparing the SCP amplitudes and latencies induced by the subcortical vs. cortical lesions and paretic vs. healthy hand movements. We used the healthy hand movement related SCPs as reference because it is related to healthy motor output. Although brain activity might not be the same as in a healthy person, the motor output is normal. Due to the severity of motor impairment in our participants, we expected to observe widespread (i.e., bilateral) SCP activity and earlier SCP onset in paretic compared to the healthy hand movements (compensatory movement planning). An ipsilateral over-activation (i.e., higher negative amplitudes) and a contralateral lower activation were expected during motor preparation of the paretic compared to healthy hand movements (maladaptive higher involvement of the intact hemisphere). Furthermore, we hypothesized that frontal and premotor areas in the participants presenting mixed lesions (subcortical and cortical) would show higher levels of activation, due to increased compensatory efforts of the secondary motor areas, compared to the participants with subcortical lesions.

## Materials and methods

### Participants

Twenty hemiparetic (none of the participants had bilateral lesions) participants 51.4 ± 11.1 years old and 5.9 ± 5.5 years since stroke participated in the study. Ten participants (5 male, 5 female) presented subcortical lesions (Sub-L) only and 10 participants (7 male, 3 female) presented mixed lesions (Mix-L) (subcortical and cortical areas). Selection criteria were no residual finger extension and time since stroke of at least 12 months. The degree of functional severity was measured using a modified version of the Fugl-Meyer Assessment (FMA) scale. (for participant information and detailed selection criteria see the Supplementary Information). The study was conducted at the University of Tuebingen, Germany. Informed consent was obtained from all participants involved. The study was approved by the ethics committee of the Faculty of Medicine of the University of Tuebingen (Germany).

### Data acquisition

Participants underwent a 16-channel EEG recording (Acticap, BrainProducts GmbH, Germany) session [Fp1, Fp2, F3, Fz, F4, T7, C3, Cz, C4, T8, CP3, CP4, P3, Pz, P4, Oz, AFz (Ground) and FCz (Reference)]. Surface electromyographic (EMG) activity was recorded from both arms using eight bipolar Ag/AgCl electrodes from Myotronics-Noromed (Tukwila, WA, USA) on four different muscle groups (extensor carpi ulnaris, extensor digitorum, external head of the biceps and external head of the triceps) in order to detect movement onset and involuntary muscle contractions. Electrooculography (EOG) recordings were also carried out for ocular corrections.

Participants performed an audiovisual task. The imperative cue was visual (an arrow pointing right or left appearing on the screen for 5 s) and auditory (a sound indicating right or left) given concurrently. This protocol was tried to resemble movements during a standard rehabilitation session. Participants either executed a hand opening and closing movement with their healthy hand (HM) or tried to open and close the paretic hand (PM) at a comfortable personal pace for 5 s according to the audiovisual imperative cues. Participants were trained and instructed to avoid compensatory movements during the intention to open and close the paretic hand. The inter-trial-interval was randomized between 3 and 4 s and a fixation cross appeared on the screen during this inter-trial resting interval. The data acquired in one session, which was lasted around 30 min.

### Data analysis

Data were analyzed using Brain Vision Analyzer 2.0 signal processing software (BrainProducts GmbH, Germany). During the EEG preprocessing a 50 Hz notch filter was applied. Data were separated from ocular artifacts using the Gratton and Coles method (Gratton et al., 1983). Participants performed 68 trials per condition (resting, healthy and paretic hand movements). After the artifact eliminations the mean number of epochs averaged was 58 and 46, for the healthy hand and paretic hand, respectively.

We used current source density (CSD) to analyze reference free data. Although 16 channels might not be sufficient to accurately estimate the CSD, we assume that the configuration used in this study permitted the CSD calculation in the channels used in the analysis (C3, F3, Cz, Fz, C4, F4: for more on CSD method see the Supplementary Information). EEG data were filtered between 0.1 and 2.5 Hz to detect SCPs. Data were segmented from −2500 to 2000 ms, aligned to the EMG onset and to the cue onset separately. The first 500 ms of each segment were used for baseline correction.

Left- and right-sided lateralized scalp sites were swapped in the participants with the right hemispheric lesion, in order to be able to make statistical comparisons between all patients' lesioned hemisphere and intact hemisphere data (e.g., F3 for the left lesions and F4 for the right lesion were pooled) (Rosahl and Knight, 1995). Thus, in this text, when we mention paretic movements, this refers to right hand movement and when we mention healthy hand movements, this refers to left hand movements. For paretic hand movement condition, F3 and C3 will be contralateral and F4 and C4 will be ipsilateral to the movement and for healthy hand condition F3 and C3 will be ipsilateral and F4 and C4 will be contralateral (Table 1).

**Table 1:** **Referred channels according to the conditions**.

	**Healthy hand movement (HM)**	**Paretic hand movement (PM)**
	**(Left hand)**	**(Right hand)**
Contralateral	(Intact/right hemisphere)	(Lesioned/left hemisphere)
	C4	C3
	F4	F3
Ipsilateral	(Lesioned/left hemisphere)	(Intact/right hemisphere)
	C3	C4
	F3	F4

Six frontal and central electrodes were used for statistical analysis (F3, Fz, F4, C3, Cz, C4) because the activity of fronto-central cortices is the major source for SCPs (Deecke et al., 1969; Libet et al., 1982). SCP onset time (Onset) and peak amplitude (Peak-Amp) features were extracted, while lesion (subcortical and mixed) location and hand movement condition (paretic and healthy) were independent variables. One-Way ANOVA for group comparisons and repeated measures ANOVA with paired *t*-test *post-hoc* analysis were carried out (for more on statistical analysis see the Supplementary Information).

### EMG analysis

EMG data were processed and used to detect muscle contraction and to align segmented EEG data to muscle activity (for details see the Supplementary Information).

## Results

In order to test our different hypotheses we performed separate statistical analyses having; (A) all participants together in one group and (B) dividing them in two groups depending on the lesion location (subcortical and mixed). These analyses were performed using two features extracted from SCPs (peak amplitude and onset time) (Table 2).

**Table 2:** **Mean of SCP onset time in ms and peak amplitude in μV for subcortical and mixed lesioned participants are presented**.

**Features**	**Healthy hand movements (HM)**						**Paretic hand movements (PM)**					
	**C3**	**F3**	**Cz**	**Fz**	**C4**	**F4**	**C3**	**F3**	**Cz**	**Fz**	**C4**	**F4**
Sub-L	−111	−267	−127	−327	−222	−347	−754	−921	−747	−889	−710	−898
Onset												
Sub-L	−4.5	−8.8	−9.4	−12.7	−9.4	−12.1	−12.9	−12.6	−16.5	−15.1	−11.1	−10.6
Peak−Amp												
Mix-L	−166	−200	−187	−185	−172	−197	−722	−805	−692	−748	−724	−752
Onset												
Mix-L	−5.8	−9.3	−9.3	−12.3	−8.3	−11.4	−8.9	−10	−15.9	−15.6	−13.7	−14.6
Peak−Amp												

### All participants

**Figure 1:**
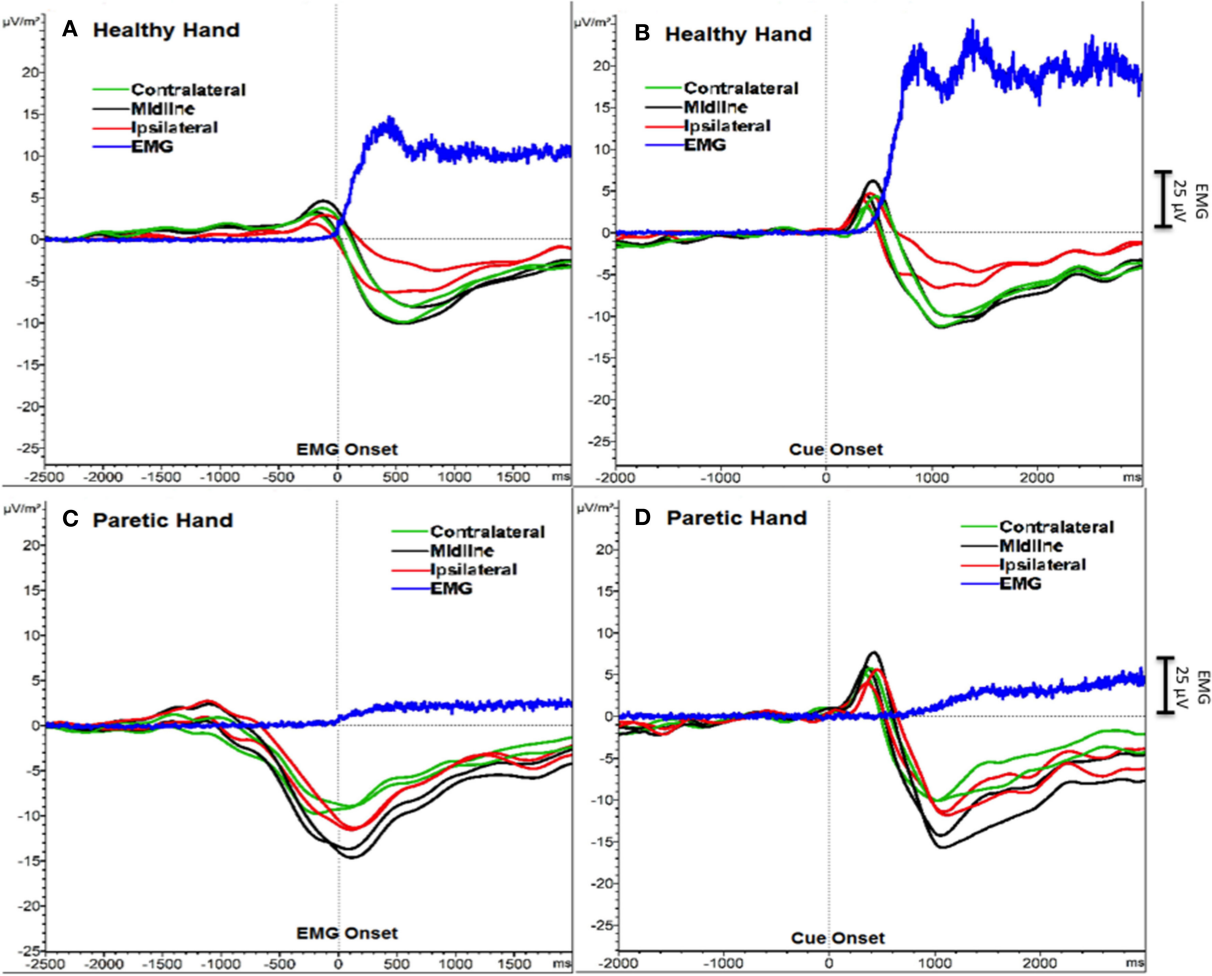
**SCPs of severe chronic stroke patients averaged and aligned to EMG onset (A, C) and to cue onset (B, D)**. The Y-axis represents SCP amplitude (μV/m^2^) and the X-axis represents time (ms). An additional Y-axis bar (25 μV) at the right side represents the EMG amplitudes.

We detected the two components of SCP (BP and MP) as one transient response occurring as a negative slope and a following peak. Figure 1 shows the grand averages of PM (paretic “right” hand) and HM (healthy “left” hand) aligned to the EMG onset and cue onset (time 0) separately. Mean EMG onset for PM was 1380.6 ± 453.3 ms and for HM was 615.7 ± 147.6 ms after the cue onset. In order to avoid this reaction time delay between PM and HM, which may influence the SCP analysis, we analyzed further EEG data aligned to the EMG onset only, not to the cue onset.

Additionally, a positive peak was clearly observed after the cue onset as a P300 potential (Polich, 2007) reflecting orienting to the stimuli (Figure 1). However, we did not include this P300 response into our analysis and we did not compare the results aligned to the cue onset, because data aligned to the cue onset would have resulted in biased SCP amplitude and latencies.

#### Peak amplitude

The repeated measures ANOVA analysis on peak amplitude showed a significant hand effect (PM vs. HM) [*F*_(1, 19)_ = 10.19, *p* < 0.01], laterality effect [*F*_(1, 19)_ = 18.41, *p* < 0.001], hand × laterality interaction [*F*_(1, 19)_ = 3.28, *p* < 0.05], hand × fronto-central distribution interaction [*F*_(1, 19)_ = 15.55, *p* < 0.001], laterality × frontocentral distribution interaction [*F*_(1, 19)_ = 9.50, *p* < 0.01] and hand × laterality × frontocentral interaction [*F*_(1, 19)_ = 8.27, *p* < 0.01].

**Figure 2:**
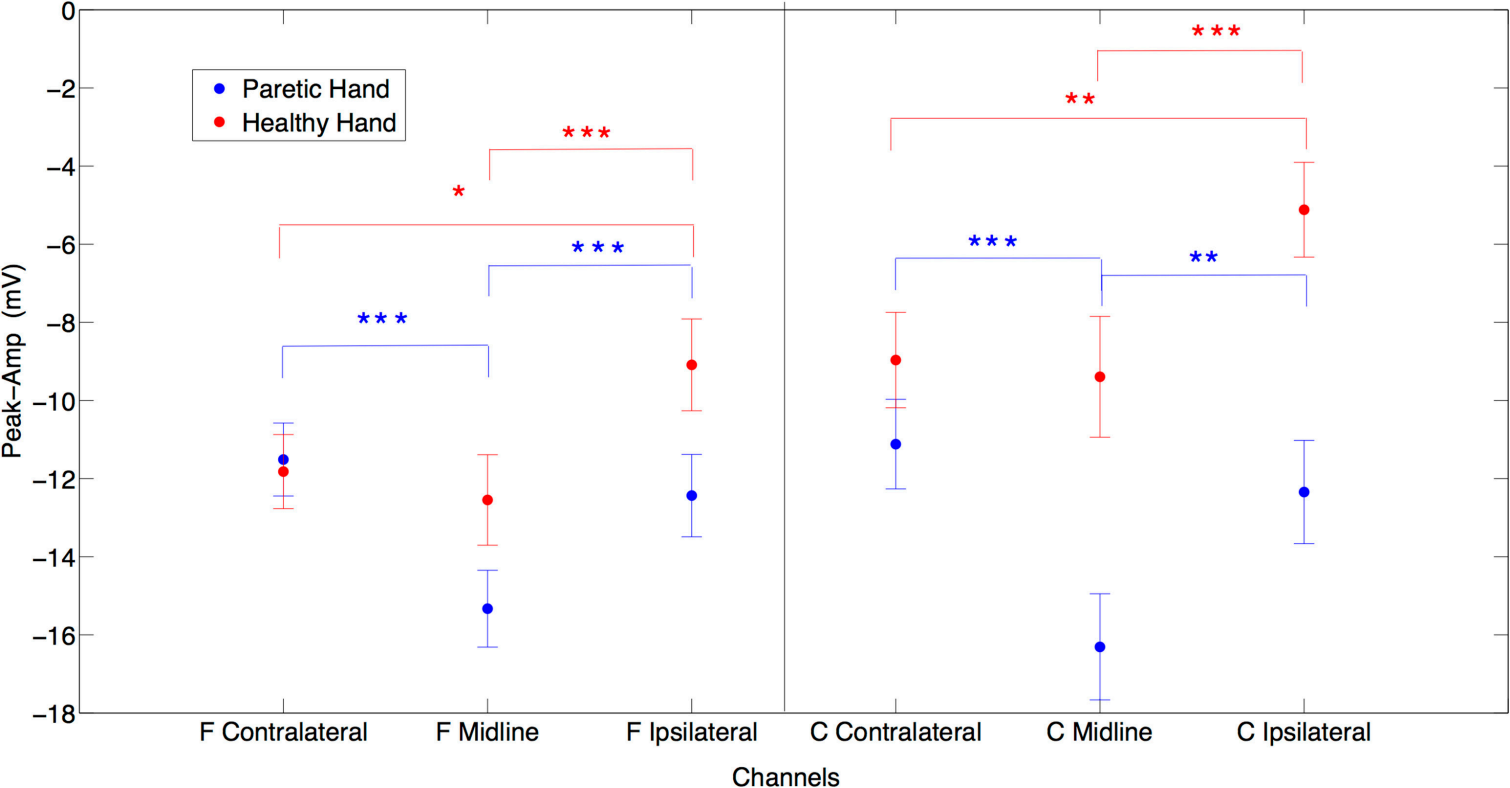
**The laterality effects on peak amplitudes in μV for both paretic (in blue) and healthy (in red) hand movements**. X axis represents frontal contralateral (F contra), frontal midline (F mid), frontal ipsilateral (F ipsi) and central contralateral (C contra), central midline (C mid), central ipsilateral (C ipsi) channels. (^*^p = 0.05; ^**^p = 0.01; ^***^p = 0.001) (Error bars represent the standard error).

In between conditions (PM vs. HM), *post-hoc* paired *t*-test analysis showed no significant difference for peak amplitude comparing contralateral potentials during HM and PM. However, over the midline electrodes peak amplitudes were significantly larger during PM compared to HM {Fz paretic vs. Fz healthy [*t*_(20)_ = −2.16, *p* < 0.05]; Cz paretic vs. Cz healthy [*t*_(20)_ = −11.33, *p* < 0.001]}. Comparing ipsilateral activities during PM and HM, peak amplitude was significantly larger on central electrode site during PM {C4 paretic vs. C3 healthy, [*t*_(20)_ = −3.89, *p* < 0.001]} (Figure 2).

When comparing laterality factor (contralateral, midline, ipsilateral activity) within the hand movement conditions (PM and HM) we observed that in PM, Cz amplitude was significantly larger than C3 and C4 {Cz vs. C3 [*t*_(20)_ = −6.08, *p* < 0.001]; Cz vs. C4 [*t*_(20)_ = −5.89, *p* < 0.001]} and C3 and C4 amplitudes were not significantly different from each other [*t*_(20)_ = −0.9, ns]. The same pattern of activity was observed at frontal electrodes. Fz showed significantly larger amplitudes than F3 and F4 {Fz vs. F3 [*t*_(20)_ = −5.51, *p* < 0.001]; Fz vs. F4 [*t*_(20)_ = −4.06, *p* < 0.001]}. There was no significant difference between F3 and F4 [*t*_(20)_ = 0.71, ns]. This result indicates no clear laterality in PM. On the other hand, during HM (left hand) a clear lateralization toward contralateral and midline regions was observed, as expected. C4 an Cz showed significantly larger amplitude than C3 {C3 vs. C4 [*t*_(20)_ = 3.17, *p* < 0.01]; C3 vs. Cz [*t*_(20)_ = 5.43, *p* < 0.001]} and there was no significant difference between Cz and C4 (*t*_(20)_ = −0.5, ns). Similarly, F4 and Fz also showed significantly larger amplitude than F3 {F3 vs. F4 [*t*_(20)_ = 2.64, *p* < 0.05]; F3 vs. Fz [*t*_(20)_ = 5.86, *p* < 0.001]} and there was no significant difference between Fz and F4 [*t*_(20)_ = −1.14, ns] (Figure 2).

The frontocentral distribution revealed that during HM (left hand) all frontal locations presented larger amplitudes compared to central locations {F3 vs. C3 [*t*_(20)_ = −3.88, *p* < 0.001]; Fz vs. Cz. [*t*_(20)_ = −2.68, *p* < 0.05]; F4 vs. C4 [*t*_(20)_ = −2.71, *p* < 0.05]} while during PM (right hand) there was no significant frontocentral distribution difference (Figure 2).

#### Onset time

The repeated measures ANOVA analysis on SCP onset time revealed a significant hand effect [*F*_(1, 19)_ = 45.48, *p* < 0.001]. Further *post-hoc* analyses were applied to test the hypothesis that before PM there will be a longer preparation time comparing HM.

*Post-hoc* paired *t*-test comparisons of laterality (contralateral and ipsilateral) of SCP onset between hand movement conditions (paretic and healthy) showed that in PM, all locations' (contralateral, midline, ipsilateral) SCP onsets were significantly earlier (i.e., SCP negativity started earlier relative to the EMG onset) than in HM {F3 paretic vs. F4 healthy [*t*_(20)_ = −5.94, *p* < 0.001]; Fz paretic vs. Fz healthy [*t*_(20)_ = −5.91, *p* < 0.001]; Cz paretic vs. Cz healthy [*t*_(20)_ = −6.15, *p* < 0.001]; C3 paretic vs. C4 healthy [*t*_(20)_ = −5.82, *p* < 0.001]; F4 paretic vs. F3 healthy [*t*_(20)_ = −6.52, *p* < 0.001]; C4 paretic vs. C3 healthy [*t*_(20)_ = −6.75, *p* < 0.001]}.

*Post-hoc* paired *t*-tests revealed that the laterality effect on onset time was not significant during HM within the condition, (i.e., the activity started bilaterally and was widespread). However, during PM, onset was significantly earlier at electrode F3 only (contralateral to the movement, over the lesioned hemisphere) when compared to Fz [*t*_(20)_ = −2.27, *p* < 0.05].

*Post-hoc* analysis of fronto-central distribution of onset time revealed that during HM, similar to what was obtained in the peak amplitude analysis (larger amplitudes for frontal regions), F3 and Fz showed also significantly earlier onset of SCP than C3 and Cz, respectively {F3 vs. C3 [*t*_(20)_ = −2.77, *p* < 0.05]; Fz vs. Cz [*t*_(20)_ = −2.35, *p* < 0.05]}. There was no significant difference between F4 and C4 (on the contralateral (healthy) hemisphere during HM [*t*_(20)_ = −1.76, ns]. These results indicate that in HM, SCP started significantly earlier in frontal regions compared to central locations over the midline and ipsilateral (lesioned) hemisphere. Additionally, all frontal regions presented significantly earlier SCP onset compared to central regions during PM {Fz vs. Cz [*t*_(20)_ = −2.17, *p* < 0.05]; F3 vs. C3 [*t*_(20)_ = −3.13, *p* < 0.01]; F4 vs. C4 [*t*_(20)_ = −2.78, *p* < 0.05]}.

### Subcortical vs. mixed lesioned participants

**Figure 3:**
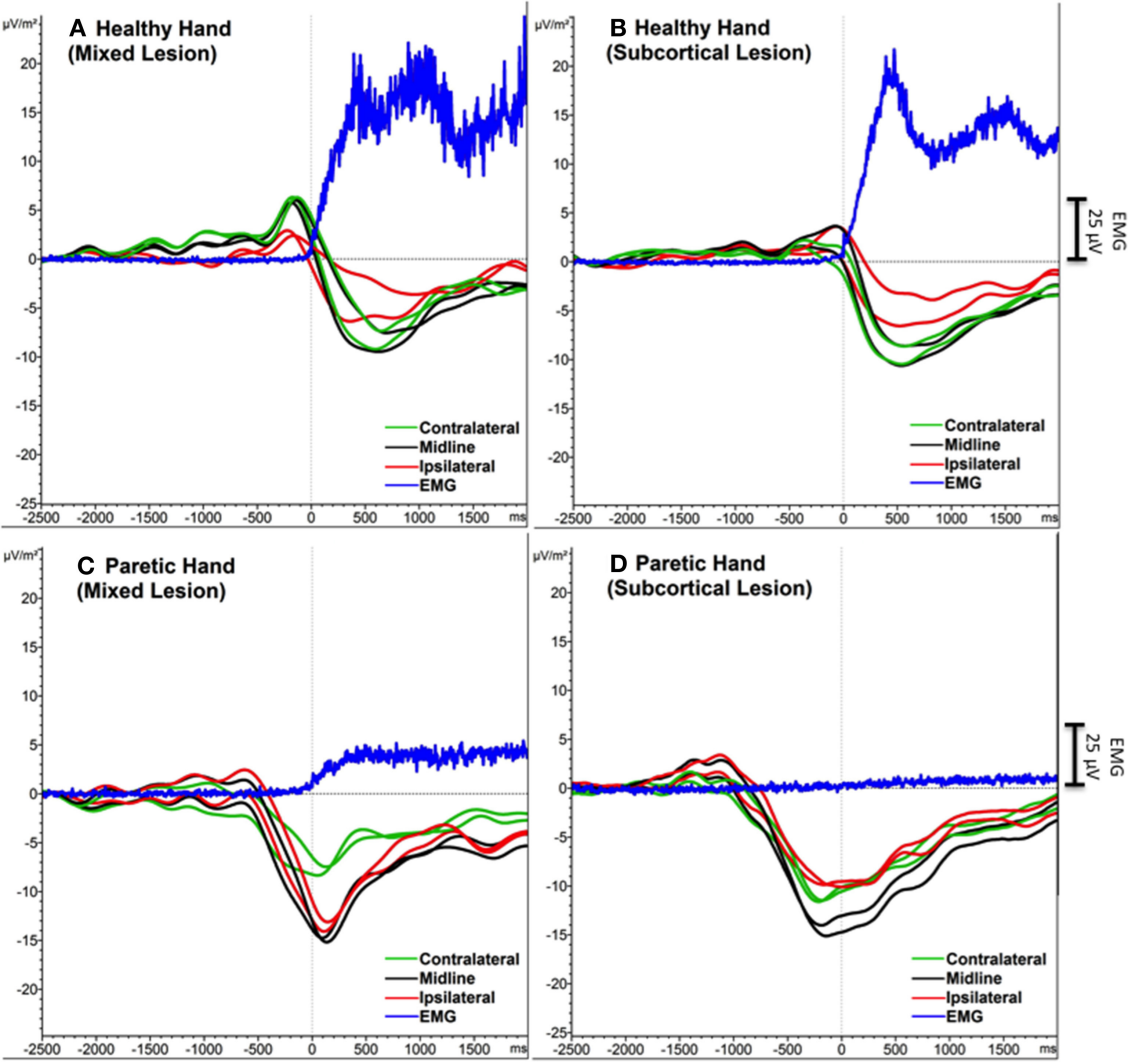
**Grand average SCPs of the participants with mixed (subcortical and cortical) lesion (A, C) and with subcortical lesion (A, D)**. Contralateral (green), midline (black) and ipsilateral (red) electrodes were shown for each movement condition. EMG activity is in blue. An additional Y-axis bar (25 μV) at the right side represents the EMG amplitudes.

FMA score difference between subcortical and mixed lesion groups was not significant [*F*_(1, 19)_ = 3.39, ns]. One-Way ANOVA analysis did not show any difference between Sub-L and Mix-L group of participants for any of the extracted SCP features (Peak-Amp and Onset), neither in PM nor in HM (Figure 3).

## Discussion

The two SCP components (BP and MP) concatenated as a NS peaking around movement onset. During PM we observed the negative peak before the EMG onset and during HM after the EMG onset. The NS started around 1 s before the EMG onset (i.e., SCP mean onset was −822 ± 338 ms) and the peak was observed around 120 ms after the EMG onset for PM. For HM the NS started around the time of the EMG onset (i.e., SCP mean onset was −212 ± 236 ms) and the peak was observed around 500 ms after the EMG onset. Although we believe our EMG onset calculation is robust, one limitation of our study could be the EMG onset difference between groups. Six out of the 10 patients belonging to the subcortical group presented no EMG onset in the paretic hand and our extrapolation method, using a constant calculated on all patients from both groups presenting EMG onset, may be inappropriate.

The SCP NS started significantly earlier and lasted longer in preparation for PM than HM and negligible preparation time was detected before HM, indicating a significantly longer movement preparatory phase during PM. We assume earlier SCP onset represents a longer neural computational effort to evoke the necessary brain excitation to induce a motor top-down command during PM. This result supports previous findings suggesting that the time between the onset of the SCP and the EMG onset may indicate extended preparation (Tarkka and Hallett, 1990)—the brain starts exciting motor networks (facilitating the neural firing) for a significantly longer time before an EMG onset can be produced on the paretic side compared to healthy hand motor actions.

Longer planning time was needed for PM. Individuals with severe paralysis lack contingencies between volitional actions and consequences. Such contingencies cannot therefore be used to drive reorganization within functional brain networks. Consequently, such individuals are prone to devolution toward a maladaptive state indicative of learned disuse (Taub et al., 1999; Krakauer, 2006; Pomeroy et al., 2011). Therefore, motor intention contingency delivered by a BMI driven orthosis may explain the positive results of Ramos-Murguialday et al. work and should reduce the time lapse between SCP peak and EMG onset. Furthermore, a combination of SMR and SCP feedback could improve BMI feedback optimizing the rehabilitation intervention.

Due to the severity of motor impairment in our participants we expected to observe compensatory over-activation (i.e., larger peak amplitudes) during motor preparation for execution attempts of PM compared to HM generated SCPs. The peak amplitude of SCP was significantly larger during PM over the midline (Fz, Cz) and ipsilateral central (C4) locations than during HM. It has been previously suggested that the amplitude of the negativity indicates the brain's computational demand to perform the movement (Jankelowitz and Colebatch, 2005). Therefore, our results denote a higher effort during PM. However, this higher activity occurs over ipsilateral central areas and medial-fronto-central areas, which may be due to maladaptive (since participants did not recover) over-activation of the healthy hemisphere and/or to the lack of cross-hemispheric inhibition from lesioned to non-lesioned hemisphere.

On the other hand, between conditions we did not observe any significant difference in SCP amplitude at the electrodes contralateral to the movement during PM (C3) compared to HM (C4). We expected a decrease in amplitude of SCPs due to the lesion in the contralateral (ipsilesional) hemisphere during PM but this was not the case. Cortical generators of SCP might not be compromised and the recorded SCP amplitude during PM could remain at similar levels if the SCP generated in subcortical structures does not contribute significantly (due to volume conduction) to the activity recorded with the EEG electrodes.

We observed that preparation of movement comes from frontal and not from central areas. The larger response on the healthy side could reflect the non-use or atrophy of the lesioned side or it may indicate a compensatory over-activation on the healthy side.

Following previous findings (Kitamura et al., 1996) we did not find any difference in peak amplitude between contralateral (ipsilesional) and ipsilateral (contralesional) hemispheres during PM, while during HM the amplitude was significantly larger in contralateral compared to ipsilateral electrodes (Green et al., 1999). Since peak amplitude has been related with the computational demand, one could expect either an increase in the peak amplitude on the ipsilesional side if cortex is intact (subcortical lesion), which is what happened in all participants, or a decrease in peak amplitude in primary motor areas and an increase in other non-lesioned secondary motor areas if cortex is lesioned (mixed lesion). We hypothesized that frontal and premotor areas in participants presenting mixed lesions (subcortical and cortical) would be more activated, due to the compensatory role of the secondary motor areas to overcome the loss of primary motor cortical structures in the participants presenting cortical lesions. However, we did not observe any significant difference between Sub-L and Mix-L groups, neither in the peak amplitude nor in SCP onset during PM or HM. This may be due to the fact that in participants presenting mixed lesions, the cortex was not heavily affected and peri-lesional areas could induce similar SCP peak amplitudes. However, since the top-down control of the motor neurons is not efficient and there is no afferent return confirming the delivery of the efferent signal, other areas of the cortex (medial and ipsilateral) are excited (facilitation of neural firing) to exploit their connection to the motor neurons of the affected muscles, resulting in significantly higher SCPs amplitudes and in maladaptive compensatory functional brain changes.

## Conclusion

We describe SCP changes in chronic severe stroke patients. Paralyzed participants showed significantly longer movement planning time and significantly larger SCP peak amplitudes in medial and ipsilateral sites during PM, indicating larger computational demand during paretic hand intention to move. Non-use of the paretic hand induces over-activation of the healthy hemisphere with larger SCP amplitudes and, probably, stronger maladaptive inhibition of the lesioned side impeding cortical reorganization and motor rehabilitation. These changes were independent of cortex integrity. SCP latency and peak amplitude in both hemispheres appear to be appropriate features to be used and optimized in BMI-like neurorehabilitation interventions.

### Conflict of interest statement

The authors declare that the research was conducted in the absence of any commercial or financial relationships that could be construed as a potential conflict of interest.
