# Supplementary material for: Movement related slow cortical potentials in severely paralyzed chronic stroke patients
Source: Front Hum Neurosci. 2015 Jan 15;8:1033. doi: 10.3389/fnhum.2014.01033 (PMC4295525; doi:10.3389/fnhum.2014.01033)
Supplement: Supplementary file 1 [file DataSheet1.DOCX]

***Supplementary Material***

Movement related slow cortical potentials in severely paralyzed chronic stroke patients

Ozge Yilmaz PhD*^1,2^, Niels Birbaumer PhD^1,3^ and Ander Ramos Murguialday PhD^1,4^

1 Institute of Medical Psychology and Behavioral Neurobiology, University of Tuebingen, Germany

2 Department of Psychology, Brain and Mind Studies Lab, Bahcesehir University, Istanbul, Turkey

3 Ospedale San Camillo, Istituto di Ricovero e Cura a Carattere Scientifico, Venezia—Lido, Italy

4 Health Technologies Department, Tecnalia, San Sebastian, Spain

*** Correspondence:** Ozge Yilmaz, PhD, Department of Psychology, Bahcesehir University, Ciragan Cad. 2-4 34353 Besiktas, Istanbul, TURKEY, Tel:0090 212 3815331, Fax: 0090 212 3810300

[ozge.yilmaz@eas.bahcesehir.edu.tr](mailto:ozge.yilmaz@eas.bahcesehir.edu.tr)

1. **Supplementary Methods**
2. Participants:

Participant selection criteria (Ramos-Murguialday et al., 2013):

- age between 18 and 80 years;
- no psychiatric or neurological condition other than stroke;
- no bilateral cerebellar lesion or bilateral motor deficit;
- no pregnancy;
- no epilepsy or medication for epilepsy during the last 6 months;
- ability to understand and follow instructions (IQ above 80 (standard progressive matrices),

no psychological impairment (self-rated by patients or relatives, physician information letter e.g.

medication or screening));

- all patients presented Mini-Mental-State-Test scores above 23;
- Fugl-Meyer Assessment (FMA) based on motor skills of hand and fingers score 12 points or below (maximal score is 24 points);
- neither aphasia nor neglect-affected patients were accepted.

Since our patients had chronic stroke, the handedness was not checked using a handedness scale. Because after such time with a paretic limb, even if the healthy hand was not dominant becomes dominant. Therefore, we did not consider handedness as a differential factor. However, in order to define the subjects’ dominant hand, the “Hand-Dominanz-Test” [Steingrüber and Lienert, 1971] was applied (See Table 1).

B. Data Analysis:

CSD is a spatial filtering technique reducing the redundancy and ambiguity of volume conduction measures in EEG (Tenke et al., 2011). Using these surface Laplacian methods like CSD decreases the contribution of the muscle artifacts to the signal (Fitzgibbon et al., 2013), which is one of the main problems with the patients who have strong movement compensation urge. The CSD method used was embedded in the EEG analysis program (Brain Vision analyzer) of Brain Products GmbH (Germany). The order of splines was 8 and maximal degree of polynomials was 10.

Under the assumption of an isotropic scalp representation, a surface Laplacian is proportional to the radial component of the gradient of the scalp current density (Nunez, 1981), also called more shortly ‘scalp current density’ (SCD; Perrin et al., 1989) or ‘current source density’ (CSD; Nunez et al., 1994). The surface Laplacian is a reference free method (Nunez, 1981; Pernier et al., 1988) acts as a high-pass spatial filter and removes the blurring effect of the diffusion of the currents through the highly resistive skull and tissues (Nunez, 1981). Tandonnet et al (2005) demonstrated that hat local and global methods of surface Laplacian estimation are equivalent to improve the spatial resolution of EEG traces. Global methods allow exploring scalp topography and local methods allow sparing time in electrode setting that can be useful for studies on special populations (i.e. children, aged subjects) and for clinical purposes. Therefore, and most importantly, they demonstrated that on the basis of a priori hypotheses concerning the generators of the measured activities, only a few sites are of interest, it is not necessary to compute interpolations that require a large number of electrodes.

One-sample Kolmogorov Smirnov was used to test normality and indicated that the data were normally distributed. One-way ANOVA was used for statistical comparisons between patients groups divided depending on lesion locations (subcortical: SubL, mixed: MixL). Significances between hand movement conditions (paretic and healthy) were tested using repeated measures ANOVA (rmANOVA), which included factors of hand movement condition (2 levels: paretic, healthy) and laterality of the SCP (3 levels: contralateral, midline, ipsilateral) and c) fronto-central SCP distribution (2 levels: frontal, central). For the repeated measure ANOVA we did assume sphericity and Greenhouse-Geisser correction was used. All the post-hoc multiple comparisons (pairwise t-tests) were Bonferroni corrected.

Post-hoc evaluations were performed using paired t-tests. Pairs were compared within hand movement conditions and in between hand movement conditions to test 3 different effects:

1. Laterality effect within hand movement condition: The pairs were C3 vs. C4, C4 vs. Cz, Cz vs. C3, F3 vs. F4, F4 vs. Fz, Fz vs. F3. These comparisons were performed for paretic hand and healthy hand movements separately.
2. Fronto-central distribution effect within hand movement conditions: The pairs were C3 vs. F3, C4 vs. F4, Cz vs. Fz. These comparisons were done for paretic hand and healthy hand movements separately.
3. In between hand movement conditions (right hand is paretic and left hand is healthy): Contralateral pairs were C3 for paretic hand movement condition vs. C4 for healthy hand movement condition, and F3 for paretic hand movement condition vs. F4 for healthy hand movement condition. Midline electrodes (Cz and Fz) were paired between paretic and healthy hand movements. Ipsilateral pairs were C4 for paretic hand movement condition vs. C3 for healthy hand movement condition, and F4 for paretic hand movement condition vs. F3 for healthy hand movement condition.

C. EMG Analysis

EMG was filtered 10 to 90 Hz and rectified. EMG data from 1500 ms to 1400 ms before each cue were used to calculate a resting baseline mean and standard deviation (SD). We determined an EMG activity onset to occur when a threshold at 4 SD from baseline mean is crossed. EMG data from 300 ms to 2000 ms after the cue were used to detect the EMG onset.

EMG onset of the healthy arm was detected clearly in all participants. EMG of the paretic arm was detected in a sequential order. The algorithm would calculate first the EMG onset on the electrodes placed on the forearm. If no EMG could be detected, EMG onset at the biceps or triceps level would be controlled and used as EMG onset. If no EMG onset in any electrode could be detected, we calculated a constant based on the EMG activity of the patients presenting muscle activity to infer the EMG.

In order to set the EMG onset for the participants whose EMG activity on the paretic arm was not detected (6 participants) a constant (K) was calculated following equation 1.

 (1)

Being P_i_ paretic and H_i_ healthy EMG onset latency of patient i and N the number of patients where EMG was detected. After K was obtained, it was multiplied with the healthy hand EMG onset latency of the patients who did not have an EMG onset on the paretic hand. Equation 2 is showing how mean EMG onset was calculated for a patient k.

 (2)

The EMG onset during healthy hand movement (EMG recorded on healthy side forearm extensors) was on average 2.24 times faster than the EMG onset detected during paretic hand movement (EMG recorded on paretic side forearm extensors) on those patients presenting detectable EMG onset in the paretic side (i.e. K was 2.24). For instance, the EMG activity on the paretic arm of patient number 10 could not be detected and the mean onset for the healthy arm was 560 ms after the cue onset. Therefore the EMG onset for paretic hand movements was considered to be 1254 ms after cue presentation (1254 = (560 x 2.24)).

1. **Supplementary Figures and Tables**

## Suplementary Table

**Supplementary Table 1.** Patient data and Fugl-Meyer Assessment (FMA) (Crow et al., 2008) scores

| no. | Age & dominant hand | Sex | Lesion location | FMA  score |
| --- | --- | --- | --- | --- |
| 1 | 64, right | f | Right hemispheric mixed lesion. Frontal and parietal lobe. Superior, medial, middle and inferior frontal gyrus; pre and postcentral gyrus. | 1 |
| 2 | 50, right | m | Right hemispheric mixed lesion. Frontal and parietal lobe and adjacent white matter with multiple necrotic. Middle and inferior frontal gyrus; pre and postcentral gyrus; middle temporal gyrus. | 0 |
| 3 | 58, right | m | Left hemispheric mixed lesion. Frontal and parietal lobe. Precentral gyrus. Corona radiata, thalamus, putamen, posterior CI, claustrum, external capsule, insula. | 2 |
| 4 | 44, right | m | Left hemispheric mixed lesion. Frontal and parietal lobe and the adjacent white matter. Superior, medial, middle and inferior frontal gyrus; pre and postcentral gyrus; supramarginal gyrus. Corona radiata, head of caudate nucleus. | 3 |
| 5 | 62, right | m | Left hemispheric mixed lesion. Frontal, parietal and temporal lobe. Middle and inferior frontal gyrus; pre and postcentral gyrus. Corona radiata, external capsule, claustrum, putamen, insula. | 2 |
| 6 | 26, right | m | Left hemispheric mixed lesion. Frontal and parietal lobe. Inferior frontal gyrus; precentral gyrus. Insular cortex, external capsule, CI, head of caudate nucleus, genu, putamen, thalamus. | 1 |
| 7 | 53, right | f | Right hemispheric mixed lesion. Frontal, parietal and temporal lobe. Inferior frontal gyrus; pre and postcentral gyrus; middle temporal gyrus. Corona radiata, CI, genu, external capsule, putamen, thalamus. | 1 |
| 8 | 68, right | m | Right hemispheric mixed lesion. Frontal and parietal lobe. Inferior frontal gyrus; precentral gyrus. External capsule, putamen, thalamus, CI, genu, insula. | 3 |
| 9 | 34, right | f | Right hemispheric mixed lesion. Frontal and parietal lobe. Middle and inferior frontal gyrus; postcentral gyrus. Corona radiata, caudate nucleus, external capsule, CI, genu, thalamus, putamen, insula. | 3 |
| 10 | 68, right | f | Right hemispheric subcortical lesion. Frontal and parietal lobe. White matter of inferior frontal gyrus; pre and postcentral gyrus; supramarginal gyrus. Multiple nectrotic vesicles. Trunk of corpus callosum, head of caudate nucleus, corona radiata, putamen, external capsule, CI, genu, thalamus. | 0 |
| 11 | 51, right | f | Right hemispheric subcortical lesion. Head of caudate nucleus, CI, genu, putamen, thalamus, corona radiata, external capsule, claustrum. | 4 |
| 12 | 47, right | m | Left hemispheric subcortical lesion. Corona radiata, external capsule, putamen, posterior CI, thalamus. | 4 |
| 13 | 54, right | f | Left hemispheric subcortical lesion. Corona radiata, body of caudate nucleus, external capsule, putamen, genu, anterior CI, insula cortex. | 0 |
| 14 | 45, right | m | Left hemispheric subcortical lesion. CI, genu, external capsule, putamen, thalamus, claustrum, head and tail of caudate nucleus, corona Subcortical lesion. CI, genu, external capsule, putamen, thalamus, claustrum, head and tail of caudate nucleus, corona radiate, insula. | 2 |
| 15 | 65, right | m | Left hemispheric mixed lesion. Insular and temporal lobes, white matter of the parietal and frontal lobe, putamen, CI, interna and externa, claustrum and thalamus. | 3 |
| 16 | 49, right | m | Right hemispheric subcortical lesion. Parietal lobe. White matter of Precentral gyrus. Corona radiata, anterior CI, putamen, external capsule, thalamus, insula. | 5 |
| 17 | 52, left | f | Right hemispheric subcortical lesion. Corona radiata, head of caudate nucleus, external capsule, CI, genu, putamen, thalamus, globus pallidus, claustrum. | 9 |
| 18 | 52, right | m | Right hemispheric subcortical lesion. Corona radiata, CI, genu, thalamus, external capsule, putamen, claustrum, insula and adjacent white matter. | 11 |
| 19 | 36, right | m | Right hemispheric subcortical lesion. Head of caudate nucleus, CI, genu, external capsule, putamen, claustrum, corona radiata. | 8 |
| 20 | 51, right | f | Right hemispheric subcortical lesion. Corona radiata, external capsule, thalamus, putamen, CI, genu. | 0 |

1. **References**

Crow J.L., Harmeling-van der Wel, B.C. (2008). Hierarchical properties of the motor function sections of the FuglMeyer assessment scale for people after stroke: a retrospective study. Phys Ther. 88, 1554-1567.

Fitzgibbon, S.P., Lewis, T.W., Powers, D.M., Whitham, E.W., Willoughby, J.O., Pope, K.J. (2013). Surface Laplacian of Central Scalp Electrical Signals is Insensitive to Muscle Contamination. IEEE Trans Biomed Eng. 60, 4-9.

Nunez PL. Electric fields of the brain. New York: Oxford University Press; 1981.

Nunez PL, Silberstein RB, Cadiush PJ, Wijesinghe J, Westdorp AF, Srinivasan R. A theoretical and experimental study of high resolution EEG based on surface Laplacians and cortical imaging. Electroenceph Clin Neurophysiol 1994;90:40–57.

Pernier J, Perrin F, Bertrand O. Scalp current density fields: concept and properties. Electroenceph Clin Neurophysiol 1988;69:385–9.

Perrin F, Pernier J, Bertrand O, Echallier JF. Spherical splines for scalp potential and current density mapping. Electroenceph Clin Neurophy- siol 1989;72:184–7 (Corrigenda Electroenceph Clin Neurophysiol, 1990 (76) 565–566).

Ramos-Murguialday, A., Broetz, D., Rea, M., Läer, L., Yilmaz, O., Brasil, F.L., et al. (2013). Brain-machine-interface in chronic stroke rehabilitation: A controlled study. Ann Neurol. 74, 100-108.

Steingrüber, H.J., & Lienert, G.A. (1971). Hand-Dominanz-Test: HDT. Verlag für Psychologie, Hogrefe.

Tandonnet C, Burle B, Vidal F, Hasbroucq T. The influence of time preparation on motor processes assessed by surface Laplacian estimation. Clin Neurophysiol 2003;114:2376–84.

C. Tandonnet, Burle B, Hasbroucq T, Vidal F. Clinical Neurophysiology 116 (2005) 18–24

Tenke, C.E., Kayser, J., Manna, C.G., Fekri, S., Kroppmann, C.J., Schaller, J.D., et al. (2011). Current source density measures of electroencephalographic alpha predict antidepressant treatment response. Biol Psychiatry. 70, 388-394.
